# Association of mortality and early tracheostomy in patients with COVID-19: a retrospective analysis

**DOI:** 10.1038/s41598-022-19567-w

**Published:** 2022-09-14

**Authors:** Armin N. Flinspach, Hendrik Booke, Kai Zacharowski, Ümniye Balaban, Eva Herrmann, Elisabeth H. Adam

**Affiliations:** 1 Department of Anesthesiology, Intensive Care Medicine and Pain Therapy, University Hospital Frankfurt, Goethe-University Frankfurt/Main, Theodor-Stern Kai 7, 60590 Frankfurt/Main, Germany; 2 Department of Biostatistics and Mathematical Modelling, Goethe-University Frankfurt, Theodor-Stern Kai 7, 60590 Frankfurt/Main, Germany

**Keywords:** Diseases, Medical research

## Abstract

COVID-19 adds to the complexity of optimal timing for tracheostomy. Over the course of this pandemic, and expanded knowledge of the disease, many centers have changed their operating procedures and performed an early tracheostomy. We studied the data on early and delayed tracheostomy regarding patient outcome such as mortality. We performed a retrospective analysis of all tracheostomies at our institution in patients diagnosed with COVID-19 from March 2020 to June 2021. Time from intubation to tracheostomy and mortality of early (≤ 10 days) vs. late (> 10 days) tracheostomy were the primary objectives of this study. We used mixed cox-regression models to calculate the effect of distinct variables on events. We studied 117 tracheostomies. Intubation to tracheostomy shortened significantly (Spearman’s correlation coefficient; rho = − 0.44, p ≤ 0.001) during the course of this pandemic. Early tracheostomy was associated with a significant increase in mortality in uni- and multivariate analysis (Hazard ratio 1.83, 95% CI 1.07–3.17, p = 0.029). The timing of tracheostomy in COVID-19 patients has a potentially critical impact on mortality. The timing of tracheostomy has changed during this pandemic tending to be performed earlier. Future prospective research is necessary to substantiate these results.

## Introduction

The Severe acute respiratory syndrome coronavirus type 2 (SARS-CoV-2) pandemic is a pending challenge for health care systems worldwide. Approximately 2.5–5% of unvaccinated patients who get infected with SARS-CoV-2 develop severe symptoms which require intensive care therapy and mechanical ventilation^1,2^. Additionally, vaccine breakthrough infections, especially in elderly and immunocompromised patients, leading to critical illness, have been observed^3–5^. The average invasive ventilation time required by critically ill COVID-19 acute respiratory distress syndrome (C-ARDS) patients is frequently reported to be 2 weeks or more^6,7^. About 30% of those patients undergo tracheostomy in the course of their treatment due to theoretical benefits of tracheostomy, including better ventilation physics (less dead space, less driving pressure and reduced resistance), reduction of sedative and analgesic therapy and better weaning possibilities^8–11^. Nevertheless, consent about optimal tracheostomy strategies does not exist.

Even in patients without COVID-19, recommendations for tracheostomy are controversial. The optimal timing of tracheostomy has been discussed extensively but the definition of clear guidelines was not possible because meta-analyses and reviews show mixed results concerning general outcome parameters of early (≤ 10 days from intubation to tracheostomy (ITT)) vs. late (> 10 days) tracheostomy^12–15^. The classification as early tracheostomy also shows a wide variability, however, an ITT ≤ 10 days is repeatedly used in reviews and meta-analyses^16–18^. While many studies show no impact of the timing of tracheostomy on mortality, some were able to show differences in duration of ventilator support and/or length of ICU stay in favor of early tracheostomy^19–25^.

In patients with COVID-19, three particular factors need to be considered: First, a potential reduction of ventilatory support and length of ICU stay may be of particular interest during times of scarcity of ICU beds/ventilators^26^. Second, the risk of aerosol exposure is imminent and delaying tracheostomy in COVID-19 patients until negative SARS-CoV-2 polymerase chain reaction or until less virus-concentration in aerosol is reasonable^27,28^. This precaution may reduce the transmission to health care workers^29^. Third, for patients with COVID-19, longer respiratory support is needed in comparison to patients with other viral pneumonia^30^.

These factors compromise the generalizability of existing data on tracheostomy to the context of COVID-19 substantially and only add to the controversy about optimal timing of tracheostomy. Another limitation is the modest data about tracheostomy in mechanically ventilated patients with COVID-19, although this procedure is performed in nearly one third of these patients^6,31^. Knowledge about this new disease has expanded rapidly since the pandemic outbreak in spring 2020. Improved resource allocation, techniques for aerosol minimization and a reassessment regarding the risk of nosocomial infections due to increased rates of vaccinated healthcare providers and/or available personal protective equipment may have reduced concerns about tracheostomies in these patients^32–34^.

However, further information on tracheostomy timing in patients with COVID-19 is desired. Further, it is worth investigating how the indication for tracheostomy was seen by intensivists over the course of the pandemic. This retrospective study aims to describe the timing of tracheostomy in patients with COVID-19 in a university-level ICU specialized on the treatment of ARDS.

## Materials and methods

The study and all methods were performed in accordance to the guidelines of the Declaration of Helsinki. The study has been approved by the institutional ethics board of the University of Frankfurt (#20-643). The requirement for informed consent from the study subjects was waived by the IRB of the University of Frankfurt due to the retrospective study design. The study has been registered to the Clinical Trials.gov Protocol Registration and Results System (NCT 05175859). Registered 03 January 2022. This manuscript was written according to the recommendations for Strengthening The Reporting of Observational studies in Epidemiology (STROBE) guidelines.

### Patient population

All patients that were admitted to the ICU of the University of Frankfurt between 03/2020 and 06/2021 were screened for inclusion in this analysis. Patients with Reverse-Transcriptase Polymerase Chain Reaction indicating a SARS-CoV-2 infection, requiring invasive ventilation and undergoing tracheostomy during their stay on ICU were included.

All patients received mechanical ventilation using an Elisa 800 (Löwenstein Medical, Bad Ems, Germany) or Hamilton G5 (Hamilton Medical, Bonaduz, Switzerland) ICU ventilator, as well as intensive care therapy according to the recurrent updated recommendations for the treatment of C-ARDS^35–37^.

### Tracheostomy

Tracheostomy was performed according to the standard operating procedure (SOP) of dilatative tracheostomy. In accordance with this SOP, we conducted tracheostomy by using a Tracoe^®^ experc Dilation Set for percutaneous tracheostomy (TRACOE medical GmbH, Nieder-Olm, Germany), non-fenestrated tracheostomy tube with a subglottic suction port. Tracheostomy was performed during continuous bronchoscopy using an Ambu^®^ aScope 4 Broncho Regular 5.0/2.2 (Ambu A/S, Ballerup, Denmark) single use bronchoscope for hygiene reasons. We used a size 8.5 to 9.0 inner diameter (ID) for men, size 7.5 to 8.0 ID TRACOE vario tracheostoma tube (TRACOE medical GmbH, Nieder-Olm, Germany), spiral-reinforced with low pressure cuff and subglottic suction for women. The definition regarding early tracheostomy as ≤ 10 days was based on the pre-published Cochrane Library systematic review by Andriolo et al.^17^. Our study project only considered patients who were eligible for tracheostomy due to their condition. Compared to studies that considered all patients, a clear difference in the observed mortality is apparent. In our study population, no primary surgical tracheostomy was required.

### Data collection

Clinical Data was obtained from the patient records. All data on the intensive care unit were recorded by a patient data management system (PDMS; Metavision 5.4, iMDsoft, Tel Aviv, Israel). Patient demographic data, laboratory results, severity scoring, Horovitz Index upon admission, days until tracheostomy [d], and mortality at ICU [%] were used for this analysis. Data collection ended with death or discharge from our ICU to either a rehabilitation facility, the referring hospital, the regular ward, or home.

### Statistical analyses

Continuous variables are presented as mean ± standard deviation and categorical variables are presented as frequencies and percentages. Time from intubation to tracheostomy and difference in mortality of early (ITT ≤ 10 days) vs. late (ITT > 10 days) tracheostomy were the main objectives of this study. We used mixed effect cox-regression models to calculate the effect of distinct variables on events and Spearman’s correlation to analyze the association between calendar time and ITT.

Statistical analysis was performed with SAS statistical software (version 9.4, SAS Institute, Cary, NC, USA) and R (R Foundation for Statistical Computing, Vienna, Austria. The R package “survival” were used. Further statistical analyses were carried out using SPSS (IBM Corp., Version 26, Chicago, IL, USA).

## Results

**Figure 1:**
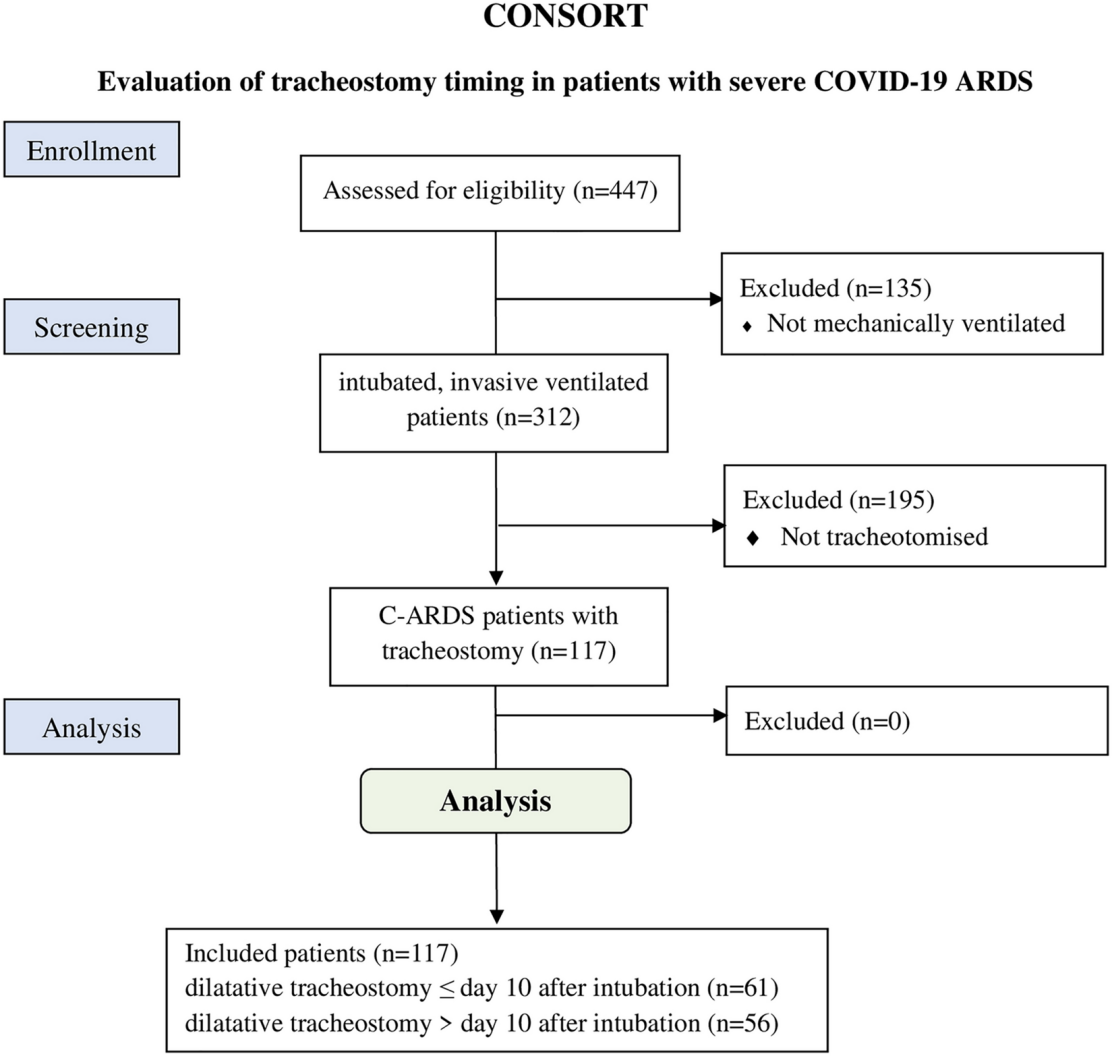
Consolidated Standards of Reporting Trials (CONSORT) diagram of patients included into the study. Diagram of the inclusion process, as well as the reasons for exclusion.

Out of 312 intubated COVID-19 patients from March 2020 until June 2021 117 (37.5%) received tracheostomy during their treatment (Fig. 1). There were no fully vaccinated patients admitted during the observation period.

**Figure 2:**
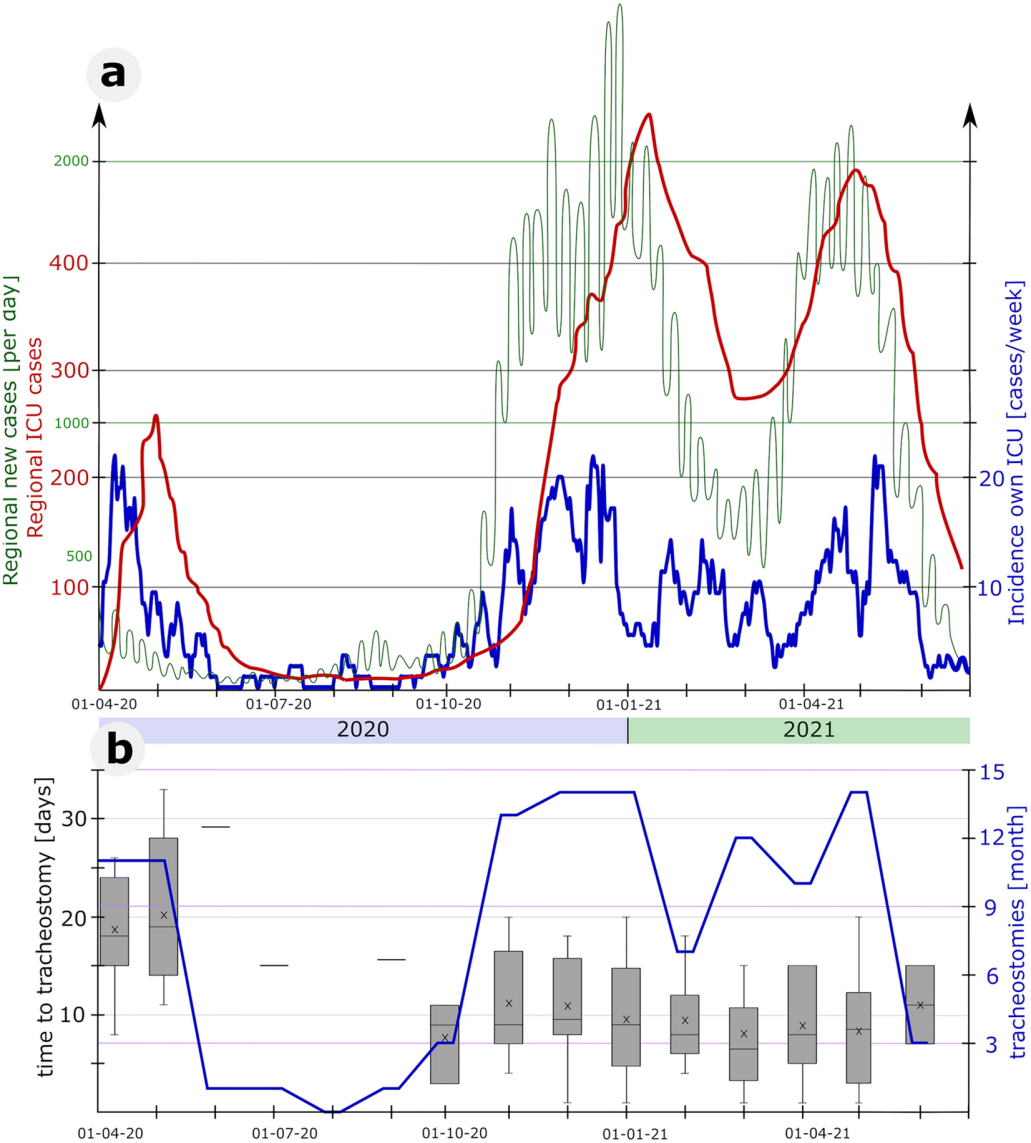
Caseload and tracheostomies through observation period. (**a**) New COVID-19 cases reported daily in the federal state of Hessen (Germany) (green) and corresponding patients treated in ICUs in Hessen (red), as well as course of own ICU admissions per week (blue)^52^. (**b**) Number of COVID-19 tracheostomies performed per month (blue) and invasive ventilation time until tracheostomy, shown as a boxplot whisker plot (grey). ICU, intensive care unit.

Out of our patient collective 61 patients (52%) underwent tracheostomy at day 10 of invasive ventilation or earlier and 56 patients (48%) after day 10. No statistical differences were observed regarding most demographic parameters and comorbidities except for diabetes and chronic kidney disease (Table 1). Inhospital mortality was significantly higher (70% vs. 43%; p = 0.003) in the early tracheostomy group. Multivariate analysis also determines tracheostomy timing as independent risk factor for in hospital mortality with an increased mortality in the early group versus the late approach (hazard ratio 1.83, CI 95% 1.07–3.17, p = 0.029) (Results of the univariate and multivariate analysis, see Table 2). In total, nine (7,7%) complications associated with tracheostomy were observed. One (0.85%) major complication needed surgical intervention, six (5.1%) minor bleedings and two (1.7%) local infections. During the observation period, months with a high rate of tracheostomy at our center were observed during high rates of new infections and ICU admissions (see Fig. 2a).

**Table 1:** Clinical characteristics of CARDS patients with tracheostomy.

Tracheostomy	Total *n* = 117 (100%)	Early ≤ 10 days *n* = 61 (52%)	Late > 10 days *n* = 56 (48%)	p-value regarding difference of groups
Mortality	67 (57.3%)	43 (70%)	24 (43%)	0.003
Time to tracheostomy (d)	11.6 ± 6.8	6.4 ± 2.8	17.3 ± 5.1	< 0.0001
Age (y)	60.1 ± 13.7	59.7 ± 14.0	60.6 ± 13.4	0.057
Sex (male)	97 (84%)	51 (84%)	46 (82%)	0.834
BMI (kg/m^2^)	32.0 ± 7.1	32.4 ± 8.1	31.5 ± 5.8	0.924
ECMO-treatment^b^	56 (48%)	34 (56%)	25 (45%)	0.233
cRRT	46 (39%)	13 (21%)	20 (36%)	0.085
SAPS II admission	47 ± 19	49 ± 19	45 ± 20	0.175
HI admission	119 ± 50	114 ± 48	124 ± 52	0.561
Coronary artery disease	32 (27%)	19 (31%)	13 (23%)	0.338
Obesity^a^	63 (54%)	32 (52%)	31 (55%)	0.754
Pulmonary disease	29 (25%)	15 (25%)	14 (25%)	0.242
Chronic kidney disease	13 (11%)	3 (5%)	10 (18%)	0.027
Diabetes mellitus	51 (44%)	32 (52%)	19 (34%)	0.044
Arterial hypertension	56 (48%)	28 (46%)	28 (50%)	0.659

Data are presented as mean ± standard deviation, count or as patient number [percentage] where applicable. Clinical characteristics of all patients, patients with early (≤ 10 days), patients with late (> 10 days) tracheostomy and corresponding p-value of differences between both time allocations.

BMI, Body mass index; d, day; HI, Horovitz index, p_a_O_2_·F_i_O_2_^–1^; kg, kilogram; m, meter; cRRT, continuous renal replacement therapy; SAPS II, Simplified Acute Physiology Score II; y, year.

^a^Defined according to international guidelines as BMI > 35 kg/m^2^.

^b^ECMO was initiated according to the current recommendations of the Extracorporeal Life Support Organization (ELSO). Thus, ECMO was usually initiated before tracheostomy.

**Table 2:** Uni- and multivariate regression mortality analysis.

	Univariate		Multivariate	
	HR (CI 95%)	p-value	HR (CI 95%)	p-value
Tracheotomy ≤ 10 vs > 10	1.93 (1.12, 3.33)	0.018	1.83 (1.07, 3.17)	0.029
coronary artery disease	1.87 (1.08, 3.23)	0.025	2.02 (1.16, 3.52)	0.014
SAPS II*	1.02 (1.01, 1.03)	0.007	1.01 (0.99, 1.03)	0.136
TISS 28 score*	1.04 (1.02, 1.07)	0.002	1.04 (1.01, 1.07)	0.011
Horovitz*	0.99 (0.99,1.00)	0.017	0.99 (0.98, 1.00)	0.002
ARDS severity*	0.65 (0.42, 1.03)	0.066		

Reproduction of the univariate and multivariate analysis of mortality in terms of the designated variables, including the confidence intervals and hazard ratio.

ARDS, acute respiratory distress syndrome; CI, confidence interval; HR, hazard ratio; SAPS II, simplified acute physiology score; TISS 28, therapeutic intervention scoring system.

* upon ICU admission.

Inhospital mortality in tracheostomized patients was 57.3% (n = 67), 47.9% (n = 56) received extracorporeal membrane oxygenation (ECMO) therapy and 39.3% (n = 46) received continuous renal replacement therapy. The most frequent observed comorbidities were arterial hypertension and obesity in 47.9% (n = 56) and 53.8% (n = 63) of patients. Existing coronary artery disease was noted in 32 patients (27.4%) of our cohort. Patient characteristics are summarized in Table 1.

Patients underwent mechanical ventilation for a mean time of 30 ± 19 days (median 27 days; interquartile range [IQR]_1–3_ 18–40). Mean ITT was 11.59 ± 6,78 days (median 11 days, IQR_1-3_ 7–15). Detailed ITT per month is presented in Fig. 2b. The data shows a reduction in ITT over the course of the pandemic with 18.64 ± 5.46 days (median 18 days; IQR_1-3_ 15.5–22.5) in April 2020 and 8.91 ± 4.93 days (median 8 days; IQR_1-3_ 3.5–11.75) in April 2021. Spearman’s correlation coefficient shows moderate negative correlation (rho = -0.44 [p ≤ 0.001]) of ITT and course of the pandemic.

SAPS II, TISS-28, Horovitz-index and degree of ARDS upon admission were associated with higher mortality in all tracheostomized patients (p = 0.007, p = 0.001, p = 0.002 and p = 0.017). Further, coronary artery disease was associated with increased mortality (p = 0.025).

## Discussion

Tracheostomy timing in the ICU is a controversial topic with COVID-19 adding an additional layer of complexity to the discussion. We herein present data on tracheostomy timing in 117 patients with COVID-19, indicating a twofold increase in mortality when tracheostomy is performed at day ten or earlier. Mortality and demographics in all of our patients are similar to general data of critically ill patients with COVID-19^31,38^. With a mean invasive ventilatory time of 30 days on our ICU and a tracheostomy rate of 37% in our cohort, our data support the notion that patients with C-ARDS have an increased duration of respiratory support compared to patients with other virus-induced ARDS. Consequently, a high proportion of these patients undergo tracheostomy^39,40^. Assessing our data from the first wave, we observed long ITTs of 18.64 ± 5.46 days in April 2020. Similar data can be observed in the Netherlands, for example, which suggest that tracheostomy was performed exceptionally late during the first wave of the pandemic from March to May 2020 in comparison to standard practice^41^. This observation may be explained by particular concerns of infection of healthcare providers when performing tracheostomy at the beginning of this pandemic which resulted in recommendations by many medical societies to delay this aerosol generating procedure^28^. Interestingly, we observed a significant change in the timing of tracheostomy during the course of this pandemic and ITT was reduced by approximately 50% between April 2020 an April 2021 (18.64 days to 8.91 days). Possible reasons for this change may be:

1. Data suggesting that risk of nosocomial infection is not as high as initially believed^39^
2. More trust in the then ubiquitously available personal protective equipment
3. Techniques for minimizing aerosol generation^32–34^
4. Vaccination of tracheostomy-performing intensivists
5. internal training for aerosol reduced approach
6. effective infection prevention and control

As described by McGrath et al., tracheostomy has changed from a reserved approach to “business as usual”^42^.

This change in timing led to two homogenous groups of patients undergoing early and late tracheostomy at our center with similar sample sizes (61 early vs. 56 late tracheostomies) and enabled a mortality-comparison of early vs. late tracheostomy. With an increased mortality hazard ratio when performing tracheostomy at day 10 or earlier, early tracheostomy appears to worsen patient outcome in COVID-19. This is rather surprising, as we expected a benefit or at least non-inferiority of early tracheostomy for our patients, because studies in patients with or without COVID-19 infection scarcely indicated increased mortality in patients undergoing early tracheostomy^12–18,41,43^. Our data appear to indicate that delaying tracheostomy may in fact benefit our patients. The observed higher mortality in patients undergoing early tracheostomy in our study population contradicts studies investigating mortality rates related to the timing of tracheostomy in pre-pandemic patients. This may be due to the fact that all patients we included had severe CARDS. In this respect, the cohort we observed showed a homogeneity with regard to the aetiology of mechanical ventilation that is otherwise difficult to find. A direct comparison of our COVID-19 cohort with patients mechanically ventilated due to other causes is therefore difficult. Further, later in this pandemic the general treatment of COVID-19 improved significantly with the use of dexamethasone, tocilizumab and antibody therapy, for example^7,44,45^. This may emphasize our results even further as patients with early tracheostomies did benefit more often of these improved circumstances than patients from the beginning of this pandemic, whom were in the late group more frequently.

Another aspect to be considered is the mutation-related transformation of the virus wild type to the now local and globally predominant subtypes of the COVID-19 virus that occurred during the observation period. Mutations massively increased the infectivity compared to the wild type, while the disease severity and thus the number of critically ill patients decreased significantly, especially in the omicron variant, which has been prevalent since the beginning of 2022^46^. During the observation period of this study, according to the viral genome sequencing of our laboratory, patients at our center primarily presented with the wild-type, alpha, beta, and delta variants, which varied only slightly in disease severity^47^.

An explanation for this increased mortality is difficult to generate, but a possible reason could be the standard use of endotracheal tubes (ETT) with subglottic suction in all of our ventilated patients. These are proven to reduce ventilator associated pneumonia, diminishing a major benefit of tracheostomy^48^. It is uncertain, whether this was the case in other studies as the type of ETT is generally not described. Secondly, although not specifically studied for tracheostomized patients, aggravated sedation was observed in COVID-19 which might overshadow the usual reduction of sedatives leading to more spontaneous breathing and faster weaning accompanied with tracheostomy^8–11,49,50^. Therefore, hypothetically, only the trauma of the procedure with risk of complications remains if these two major benefits are taken out of the equation. Our observed complication rate (7.7%) is comparable to data from the literature^11,51^.

All put together, our study shows that tracheostomy practice in COVID-19 changed. Our reduction in ITT occurred although, neither SOPs or guidelines concerning tracheostomy-timing changed during our observation period^35–37^. We strongly believe that similar reductions in ITT occurred in other centers as well and we are now able to show the impact of this change on mortality: an earlier approach was associated with a nearly doubled risk for patients to decease. The different length of ventilatory support and tracheostomy rates in C-ARDS already show the distinction to other ARDS-types and now the best approach to tracheostomy may also be different.

Our study has some limitations that need to be considered when interpreting our findings. First, our study is a retrospective analysis of tracheostomy timing in COVID-19 patients. The comparison of early vs. late tracheostomy is therefore not based on a treatment protocol and other factors might have led to our findings. Additionally, it makes finding causes for our observation more difficult. Second, our dataset does not provide information about sedation differences and rate of pneumonia. Hence, a comparison between early and late tracheostomy is solely possible for mortality. Third, we do not provide follow-up of patients discharged to rehab, normal ward, home or the referring hospital.

## Conclusions

The timing of tracheostomy in COVID-19 patients has a potentially critical impact on mortality. In our study, a direct comparison of tracheostomy at day ten or earlier was associated with a significantly increased risk of mortality compared to a delayed procedure. Future prospective research is necessary to substantiate these results.

## Data Availability

The dataset supporting the conclusions of this article are available upon reasonable request from the corresponding author (ANF).
